# Determination of Urinary Mycotoxin Biomarkers Using a Sensitive Online Solid Phase Extraction-UHPLC-MS/MS Method

**DOI:** 10.3390/toxins13060418

**Published:** 2021-06-11

**Authors:** Jessica Schmidt, Benedikt Cramer, Paul C. Turner, Rebecca J. Stoltzfus, Jean H. Humphrey, Laura E. Smith, Hans-Ulrich Humpf

**Affiliations:** 1 Institute of Food Chemistry, Westfälische Wilhelms-Universität Münster, Corrensstraße 45, 48149 Münster, Germany; j_schm63@uni-muenster.de (J.S.); cramerb@uni-muenster.de (B.C.); 2 Maryland Institute for Applied Environmental Health, School of Public Health, University of Maryland, College Park, MD 20742, USA; pturner3@umd.edu; 3 Goshen College, 1700 S. Main Street, Goshen, IN 46526, USA; rstoltzfus@goshen.edu; 4 Division of Nutritional Sciences, Cornell University, Ithaca, NY 14850, USA; 5 Department of International Health, Johns Hopkins Bloomberg School of Public Health, Baltimore, MD 21205, USA; jhumphr2@jhu.edu; 6 Department of Population Medicine and Diagnostics, Cornell University, Ithaca, NY 14850, USA; les36@cornell.edu

**Keywords:** mycotoxin, biomonitoring, urine, online solid phase extraction, metabolite, HPLC-MS/MS

## Abstract

In the course of assessing the human exposure to mycotoxins, biomarker-based approaches have proven to be important tools. Low concentration levels, complex matrix compositions, structurally diverse analytes, and the large size of sample cohorts are the main challenges of analytical procedures. For that reason, an online solid phase extraction-ultra high-performance liquid chromatography-tandem mass spectrometry (online SPE-UHPLC-MS/MS) method was developed, allowing for the sensitive, robust, and rapid analysis of 11 relevant mycotoxins and mycotoxin metabolites in human urine. The included spectrum of analytes comprises aflatoxin M_1_ (AFM_1_), altenuene (ALT), alternariol monomethyl ether (AME), alternariol (AOH), citrinin (CIT) and its metabolite dihydrocitrinone (DH-CIT), fumonisin B_1_ (FB_1_), ochratoxin A (OTA), and zearalenone (ZEN) as well as α- and β-zearalenol (α- and β-ZEL). Reliable quantitation was achieved by means of stable isotope dilution, except for ALT, AME and AOH using matrix calibrations. The evaluation of method performance displayed low limits of detection in the range of pg/mL urine, satisfactory apparent recovery rates as well as high accuracy and precision during intra- and interday repeatability. Within the analysis of Zimbabwean urine samples (*n* = 50), the applicability of the newly developed method was shown. In addition to FB_1_ being quantifiable in all analyzed samples, six other mycotoxin biomarkers were detected. Compared to the occurrence rates obtained after analyzing the same sample set using an established dilute and shoot (DaS) approach, a considerably higher number of positive samples was observed when applying the online SPE method. Owing to the increased sensitivity, less need of sample handling, and low time effort, the herein presented online SPE approach provides a valuable contribution to human biomonitoring of mycotoxin exposure.

## 1. Introduction

Mycotoxins are secondary metabolites of fungal origin produced by various genera. The infestation of agricultural crops such as grains, fruits, nuts, or spices by filamentous fungi as well as the formation of mycotoxins is influenced by climatic factors and can occur both pre- and post-harvesting [1,2]. Because the complete removal of mycotoxins during food processing procedures is usually not possible, these contaminants are present in food- and feedstuffs [3]. As a result of carry-over effects, not only plant-based but also foodstuffs of animal origin may be contaminated [4]. Owing to multiple toxicological effects related to mycotoxins such as carcinogenicity, renal toxicity, immunosuppression, or estrogenic effects potentially occurring with chronic ingestion, the exposure assessment is of decisive importance [5]. While wealthier countries have legally specified maximum levels for contaminants in foodstuffs, especially impoverished regions are affected by dietary mycotoxin exposure [3,5].

In order to allow for the individual examination of regional, socio-economic, or alimentary differences, biomonitoring approaches are frequently used. With regard to the analytical procedures, the determination of mycotoxin biomarkers in biological fluids such as blood or urine is associated with several challenges. The low concentration levels of mycotoxin biomarkers in physiological samples require sensitive analyte detection and also the sample preparation needs to be considered as a critical point [6]. Since usual exposure situations comprise structurally diverse compounds, approaches for biomarker-based exposure assessment should be able to cover a large spectrum of analytes. Additionally, the high matrix load of body fluids complicates analyte separation and mass spectrometric detection. Accurate analysis of large sample cohorts is essential for meaningful evaluation of population exposure patterns, thus a high sample throughput capacity is also required [7]. The analysis of a high number of samples as well as at least partial reduction of matrix effects can be accomplished by using dilute and shoot (DaS) approaches, subjecting the urine samples to high-performance liquid chromatography-tandem mass spectrometry (HPLC-MS/MS) analysis after simple sample dilution [8,9,10]. This procedure has the major drawback of a reduced sensitivity due to the dilution step. More laborious approaches such as solid phase extraction (SPE), immunoaffinity chromatography (IAC), or the use of dried urine spots (DUS) facilitate analyte enrichment as well as the separation of interfering matrix compounds, resulting in lower limits of detection [11,12,13,14,15]. IAC clean-up in particular enables the specific retention of the targeted analytes, but the high selectivity makes it complex and often unsuitable for multi-mycotoxin analysis [7]. A QuEChERS-based (Quick, Easy, Cheap, Effective, Rugged, Safe) sample preparation protocol was recently used in the course of mycotoxin biomonitoring [16]. However, the method’s sensitivity could not be improved compared to DaS approaches for several compounds [8,9]. Until now, the lowest limits of detection regarding mycotoxin biomarkers in urine samples were achieved by use of a SPE clean-up procedure [13]. Nevertheless, a high sample throughput may be demanding owing to the comparatively high expenditure of time, consumption of organic solvents, and number of disposable SPE cartridges. Online SPE approaches can be appropriate alternatives to overcoming these disadvantages. Compared to offline SPE methods, the online variant exhibits major benefits such as time efficiency, reduced solvent consumption, and reusable extraction columns. Moreover, analyte loss or degradation is reduced and accuracy as well as precision can be improved due to less sample handling [17,18].

Within the scope of mycotoxin analysis, several online SPE applications considering different matrices have been developed. Besides single analyte online SPE methods for the determination of aflatoxin M_1_ (AFM_1_) in milk and dairy products [19] or zearalenone (ZEN) in beer [20], multi-analyte approaches have also been used. The analysis of aflatoxins and ochratoxin A (OTA) in dried fruits via online SPE was realized after pressurized liquid extraction [21]. For the sensitive detection of OTA and ochratoxin B (OTB) in wine, an online SPE-based procedure was developed by Kholová et al. [22]. Ates et al. applied online SPE clean-up for the determination of six *Fusarium* mycotoxins in extracts of wheat, maize, and feedstuffs [23] and a method for the analysis of 12 mycotoxins in beer samples was presented by Rozentale et al. [24]. Very recently, an online turbulent flow sample preparation method coupled with UHPLC-high resolution mass spectrometry (HRMS) for the analysis of 10 mycotoxins in human urine after glucuronidase treatment was published [25]. Also, for the analysis of target compounds like metabolites of anabolic-androgenic steroids [26] or pesticide biomarkers [27], as well as for doping control analysis [28] in urine samples, online SPE approaches proved to be suitable.

The aim of this study was to combine sensitive mycotoxin biomarker detection and short analysis times that are valuable for human biomonitoring in large sample cohorts. To that end, online SPE clean-up was applied for sample preparation and determination of 11 important mycotoxins and mycotoxin metabolites in urine samples. The spectrum of compounds included the frequently detected urinary mycotoxin biomarkers aflatoxin M_1_ (AFM_1_), citrinin (CIT) and its metabolite dihydrocitrinone (DH-CIT), fumonisin B_1_ (FB_1_), ochratoxin A (OTA), zearalenone (ZEN) as well as the metabolized forms α- and β-zearalenol (α- and β-ZEL) [29]. Moreover, the *Alternaria* toxins altenuene (ALT), alternariol monomethyl ether (AME) and alternariol (AOH) were implemented as potential biomarkers for this genus of fungi. Generally, the accurate determination of the above-mentioned compounds is mandatory to facilitate metabolite-based risk assessment of frequently occurring and toxicologically relevant mycotoxins. 

## 2. Results and Discussion

### 2.1. Online SPE-UHPLC-MS/MS Method Development

In this study, an easy and rapid online solid phase extraction-ultra high-performance liquid chromatography-tandem mass spectrometry (online SPE-UHPLC-MS/MS) method for the analysis of 11 mycotoxins and mycotoxin metabolites in urine samples is presented. Crucial points of method development included a significant lowering of the limit of detection (LOD) and limit of quantitation (LOQ) compared to frequently used approaches for the analysis of urine samples (see Table 2) while comprising short analysis times and enabling a high sample throughput. The selection of the spectrum of analytes was focused on mycotoxins most frequently occurring under hot and humid conditions, for example in South Asia or Sub-Saharan Africa [2,29]. Finally, 11 relevant mycotoxins and mycotoxin phase I metabolites, specifically aflatoxin M_1_ (AFM_1_), altenuene (ALT), alternariol monomethyl ether (AME), alternariol (AOH), citrinin (CIT) and its metabolite dihydrocitrinone (DH-CIT), fumonisin B_1_ (FB_1_), ochratoxin A (OTA), zearalenone (ZEN), α- and β-zearalenol (α- and β-ZEL) were incorporated into the validated method. 

In order to improve the sensitivity, different parameters including sample volume, mobile phase for loading and washing of the online SPE column as well as flow rate and valve switching time were investigated. Furthermore, experiments with different eluents for the chromatographic separation were carried out and the binary gradient along with the flow rate was optimized. For trapping and pre-concentration of the analytes while removing interfering matrix compounds, an Oasis^®^ HLB column (2.1 × 20 mm, 5 µm; Waters GmbH) was proven to be suitable. The Oasis HLB material has generally been shown to be able to bind mycotoxins in physiological matrices [12,13,14,30]. However, not all Oasis materials are available for online SPE applications. The injected sample volume was increased from 30 µL up to 100 µL and finally set to 100 µL in order to maximize the sensitivity for the detectable analytes. Sample preparation prior to injection comprised spiking the urine sample with a mixture of the stable isotope-labelled standards followed by centrifugation to prevent the online SPE-UHPLC system from clogging with particles. Concentrating the analytes on the online SPE column was achieved by loading and washing with H_2_O containing 0.1% of formic acid and 5 mM of ammonium acetate. For loading and washing of the online SPE column, valve switching times of 1, 2, and 3 min at a flow rate of 1 mL/min as well as valve switching times of 1 and 2 min at a flow rate of 2 mL/min were tested. Since no advantageous influence of varying flow rate and valve switching time was noticed, the urine sample was loaded onto the online SPE cartridge at a flow rate of 1 mL/min and the position of the column switching valve was changed after 2 min for analyte elution. 

With regard to the chromatographic separation on the analytical column, the use of different eluents was investigated. Like many multi-analyte methods, finding a mobile phase providing good signal intensity at low noise levels for most of the included mycotoxins and mycotoxin metabolites proved to be challenging. Due to considerably lower matrix interferences for several analytes including ZEN, α-ZEL, and β-ZEL and simultaneously good signal intensities for the other analytes, acetonitrile (ACN) and H_2_O both containing 0.1% of formic acid and 5 mM of ammonium acetate were used for the final method. The binary gradient and the flow rate were adapted for appropriate retention of the most polar analyte AFM_1_ and to allow a rapid separation within a total run time of 13 min per analysis. A short re-equilibration period of 1.5 min at the end of the total run time proved to be sufficient, since flushing of the analytical column at starting conditions was continued during loading and washing of the online SPE column. As analytical column a Nucleodur C_18_ Gravity-SB UHPLC column with a particle size of 1.8 µm (Macherey-Nagel) was chosen. The application of small particles combined with a high flow rate of 450 µL/min enabled an excellent separation efficiency in a short analysis time. Regarding the mass spectrometric detection, the multiple reaction monitoring (MRM) transitions were optimized with respect to minimize potential matrix interferences and find best signal-to-noise (S/N) ratios (see Table S1, Supplementary Material, for MRM transitions). The implementation of stable isotope-labelled standards enabled a reliable quantitation regardless of varying matrix compositions and concentrations. Nevertheless, for ALT, AME, and AOH without available internal standards, quantitation proved to be feasible by means of matrix calibrations (see Section 2.2). 

Although deoxynivalenol (DON) and its metabolites are frequently detected in urine samples, especially in Europe [29], these compounds were excluded. Using the Oasis^®^ HLB online SPE column, DON could not be separated from interfering matrix compounds so that an improvement of sensitivity compared to dilute and shoot (DaS) approaches [8,9] was not achieved. Besides, the more polar glucuronic acid conjugates were insufficiently retained on the online SPE column. The same issues were observed by Warth et al. [9] when conventional offline SPE cartridges were used. Within method development of an online turbulent flow clean-up procedure for the analysis of 11 mycotoxins and mycotoxin metabolites, DON was also excluded and analyzed using an offline SPE cartridge [25]. 

### 2.2. Method Performance

In the course of assessing method performance, an in-house validation was carried out comprising the determination of LOD, LOQ, working range, linearity, and recovery as well as intra- and interday repeatability.

Based on a S/N ratio of 3, LODs were determined in concentration ranges of picogram per milliliter urine for all analytes. For reasons of comparability, specific values are given in ng/mL. The highest sensitivity was observed for OTA, FB_1_, and AFM_1_ with LODs of 0.0036, 0.0042, and 0.0070 ng/mL urine, respectively. Calculations for ZEN, AME, α-ZEL, β-ZEL, DH-CIT, and CIT revealed values between 0.01 and 0.09 ng/mL. With respective detection limits of 0.21 and 0.27 ng/mL of urine for only ALT and AOH, concentrations in the upper picogram per milliliter scale were achieved (see Table 1). 

The working range was specified over two decimal powers from the respective LOQs, and linearity was confirmed by means of regression coefficients between 0.984 and 0.999 as well as Mandel’s fitting test. Apparent recovery rates (R_A_) were calculated with regard to matrix- and solvent-based calibrations. Consideration of the stable isotope-labelled standards resulted in R_A_ between 91% and 103% for AFM_1_, CIT, DH-CIT, OTA, and ZEN. For FB_1_ a higher R_A_ of 147% was observed, which probably can be related to a signal enhancing effect of the urine matrix compared to neat standard solutions. Determination of R_A_ for α-ZEL and β-ZEL using *d*_2_-ZEN as an internal standard led to slightly lower values of 86% and 80%, respectively. Since for ALT, AME, and AOH no stable isotope-labelled standards were available, the lowest R_A_ of 38–78% was monitored. In the case of these analytes, R_A_ represents the effect of signal suppression due to matrix interferences. Although urine concentration and composition are highly variable, matrix calibrations can be used as an alternative quantitation strategy if minor variations in the calculated concentration levels are kept in mind. 

For evaluation of intra- and interday repeatability, the method’s accuracy and precision were determined at low, medium, and high concentration levels. In order to improve accuracy and precision at low concentrations, which are expected to be found in urine samples, a 1/x-weighting of the calibrations curves was chosen. 

The intraday accuracy was calculated in a range of 95–123% at the lowest spiked concentration, except for AME and AOH. The higher deviation of the latter, yielding respective percentages of 179% and 140%, may be related to low signal intensities and the absence of the stable isotope-labelled analogues. At medium spiking levels the accuracy was observed to be even higher compared to the low concentration levels, with values of 133% for AME and 97–118% for all other analytes. The values of the interday accuracy fell into a range comparable to intraday accuracy. Solely for FB_1_ were slightly higher percentages observed regardless of the concentration level.

With respect to the precision during intraday repeatability, relative standard deviations (RSDs) between 4% and 10% were determined for AFM_1_, ALT, AME, AOH, CIT, DH-CIT, and OTA at all concentration levels. FB_1_, ZEN, α-ZEL, and β-ZEL showed slightly higher variations up to 16%, but only at low concentrations. The calculation of interday repeatability revealed RSDs of less than 11% for AFM_1_, ALT, AOH, CIT, DH-CIT, and OTA. For AME and FB_1_, values of 10–18% were observed. The highest RSDs during interday repeatability were assessed for ZEN, α-ZEL, and β-ZEL, with percentages up to 27% at the lowest spiking level. Regarding medium and high concentration levels of these analytes, the RSDs were less than 10%. Due to a very low noise level of the chosen transitions for FB_1_, ZEN, α-ZEL and β-ZEL already, signal intensities of several hundred counts per second were sufficient for the identification of these analytes. Nevertheless, the low signal intensities were related to the higher RSDs at the lowest concentration level. Accuracy and RSDs of the high spiking level are given in Table S2 (Supplementary Material).

The sensitivity of the newly developed method can be assessed by comparing the LODs to several approaches for multi-mycotoxin analysis in human urine using different types of sample preparation (see Table 2). Frequently used DaS approaches are cost-effective and minimally time-consuming. Due to the dilution of the urine samples and no removal of matrix compounds, however, higher LODs have to be accepted. The new online SPE-UHPLC-MS/MS method avoids sample dilution, removes the sample matrix, and enables introducing a higher sample volume into the analytical process, while the time effort is comparable to DaS approaches. As a consequence of the higher sample volume, LODs for AFM_1_, ALT, AME, AOH, CIT, DH-CIT, OTA, ZEN, α-ZEL, and β-ZEL were improved by factors of 3–30 compared to the DaS method based on Gerding et al. [8,10]. For FB_1_, even a 200-fold lower LOD was achieved using the online SPE method. The comparison to the DaS method of Warth et al. [9] indicated an 8–120-fold higher sensitivity of the herein presented online SPE procedure. Apart from DaS approaches, a recently published dried urine spot (DUS) procedure presents a combination of simplified sample transport and efficient sample preparation. However, sensitivity of the online SPE was not achieved with that protocol and higher LODs had to be accepted [11]. Certain multi-mycotoxin methods using more laborious sample preparation such as conventional SPE combined with immunoaffinity chromatography (IAC) [14] or liquid-liquid extraction (LLE) [30] as well as a QuEChERS-based procedure (Quick, Easy, Cheap, Effective, Rugged, Safe) [16] provided a lower sensitivity for some analytes than the presented online SPE clean-up. Nevertheless, the sole use of Oasis^®^ Prime HLB SPE columns implied considerably lower LODs [12,13].

Besides the DaS approach [8,10], the analysis of ALT, AME and AOH was only covered by a few methods for multi-mycotoxin analysis in human urine samples. Using a SPE clean-up, Liu et al. [12] determined 10–30-fold lower LODs for the aforementioned analytes compared to the results of the developed online SPE method. By contrast, a QuEChERS-based method provided comparable or higher LODs for AOH and AME, respectively, whereas ALT was not included in that study [16].

In addition to the multi-mycotoxin approaches already discussed and listed in Table 2, Ndaw et al. recently published an online sample clean-up procedure for the analysis of 10 mycotoxins and mycotoxin metabolites in urine samples [25]. While that method also incorporated AFB_1_, T-2, HT-2, and OTα, the *Alternaria* toxins as well as CIT and DH-CIT were not included. Since HRMS was used for detection, the determination of LOQ was not based on the S/N ratio but on the relative standard deviation; the LOD was not determined. The LOQ of OTA and ZEN were in the same range of concentration using the herein presented online SPE method and the turbulent flow online sample preparation of Ndaw et al. For the other analytes, the latter approach was less sensitive.

When comparing the LODs with a focus on the type of sample preparation, it should be noted that several of the cited approaches implement an enzymatic hydrolysis. The hydrolysis does not affect the LODs, but results in a higher expected amount of the respective parent compounds. Consequently, a higher number of positive samples may be observed after hydrolysis even at higher LOD values. Furthermore, the performance of the available mass spectrometer will have an impact on the sensitivity. 

With respect to the method’s precision, the offline SPE procedure of Šarkanj et al. revealed RSDs during intraday repeatability up to 20% for FB_1_ and α-ZEL, whereas a maximum RSD of 16% for ZEN was observed for the presented online SPE method. The interday repeatability of the online SPE method was reduced up to 13% compared to the offline SPE approach, except for ZEN and β-ZEL [13]. Using the other abovementioned procedures of sample preparation, comparable or even lower precisions were observed. In comparison to the sensitive offline SPE clean-up of Šarkanj et al., reduced sample handling and the automated extraction process clearly improved the precision and consequently the reliability of the calculated analyte concentrations of naturally contaminated urine samples.

### 2.3. Analysis of Human Urine Samples

**Figure 1:**
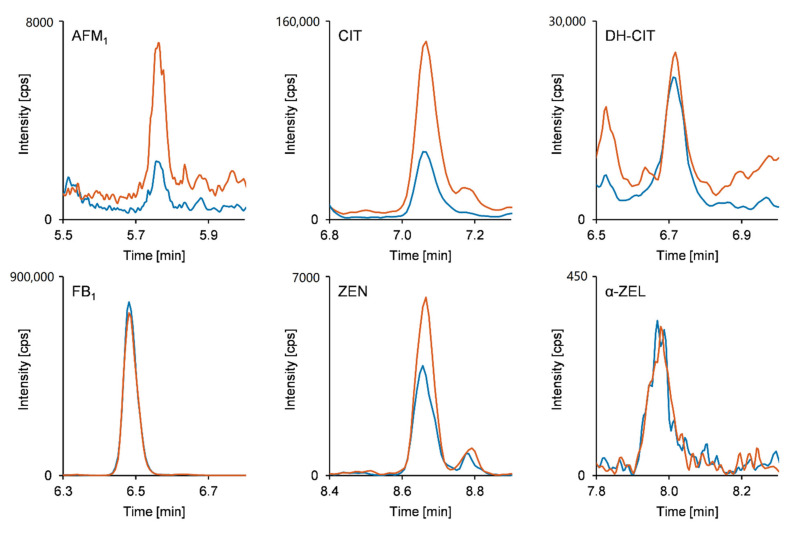
MRM chromatograms of a naturally contaminated human urine sample. Blue line: quantifier transition. Red line: qualifier transition (for details see Table S1, Supplementary Material). Calculated concentrations: AFM_1_ < LOQ, CIT 0.99 ng/mL, DH-CIT 0.30 ng/mL, FB_1_ 4.6 ng/mL, ZEN 0.27 ng/mL, α-ZEL < LOQ.

Following method validation, a subsample set of Zimbabwean urine samples from the SHINE (Sanitation, Hygiene, Infant Nutrition Efficacy) trial [31] (*n* = 50) was analyzed by employing the online SPE-UHPLC-MS/MS method in order to demonstrate its applicability. Zimbabwe is a landlocked country in Southern Africa that has a semi-tropical climate. The SHINE trial was a community-based cluster-randomized trial investigating the independent and combined effects of a water, sanitation, and hygiene intervention and a nutrition intervention on child growth and anemia. Mycotoxin exposure was additionally investigated as another potential cause of child stunting and anemia and the results for that work will be published elsewhere. The purpose of this paper is to investigate a new and improved method for measuring multiple mycotoxin exposure in urine samples. The duplicate analysis of the urine samples implied the exposure to eight out of the 11 included mycotoxins and mycotoxin metabolites (see Table 3). While FB_1_ turned out to be the most frequently detected analyte in all urine samples, the occurrence of OTA was shown in 38% of the samples followed by AFM_1_ and DH-CIT with percentages of 18% each. Detection of CIT and ZEN was achieved in 14% and 10% of the analyzed samples, respectively, whereas both AME and α-ZEL were found in only one urine sample (2%). Except for FB_1_, which was quantifiable in all samples, concentrations higher than the respective LOQ were evidenced in up to seven of 50 samples. The calculated mean urinary concentration levels ranged from 0.060 ng/mL for OTA to 1.5 ng/mL for CIT. Highest concentrations were observed for DH-CIT, CIT, and FB_1_ with respective quantitated maximum levels of 1.3, 2.3, and 4.6 ng/mL. Detailed results for all detected mycotoxins and mycotoxin metabolites are listed in Table 3. In addition to determination of the concentration levels, the co-occurrence of up to six mycotoxin biomarkers was observed (see Figure 1).

**Figure 2:**
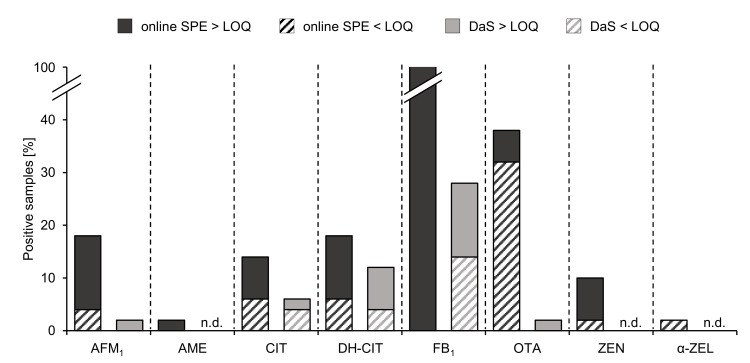
Percentages of Zimbabwean urine samples containing mycotoxins and mycotoxin metabolites determined with the developed online SPE clean-up and a frequently used dilute and shoot (DaS) approach [8,10] (*n* = 50).

Prior to online SPE-UHPLC-MS/MS analysis, mycotoxin biomarker occurrence in the Zimbabwean sample set was determined by applying a frequently used DaS approach [8,10]. The comparison of the results of both methods is summarized in Figure 2. Using the DaS method, AFM_1_ and OTA were only detected and quantified in one sample (2%). The presence of CIT was observed in 6% of the analyzed urine samples and one sample (2%) revealed a concentration above the LOQ. Detection of DH-CIT was feasible in 12% and quantitation was achieved in 8% of the samples. In compliance with the results of the online SPE method, FB_1_ proved to be the most frequently occurring analyte in 28% of the analyzed samples and exhibited a quantifiable amount of 14%. ZEN and α-ZEL were not detected. 

The comparison of the results clearly demonstrates the improved sensitivity of the newly developed method. According to the rather similar LOD for CIT and DH-CIT obtained with both procedures of samples preparation, the occurrence of these compounds was shown in approximately the same number of urine samples. The percentage of detects of AFM_1_ and OTA was considerably increased if the urine samples were analyzed by means of the online SPE approach. Moreover, the herein presented method indicated that all urine samples contained FB_1_, and the detection of ZEN and α-ZEL was also achieved due to the improved sensitivity. As depicted in Figure 2, the percentage of samples containing the analytes in quantifiable concentrations was significantly higher after online SPE-UHPLC-MS/MS analysis. These results imply that the presented online SPE method enables a more reliable risk assessment of mycotoxin exposure.

## 3. Conclusions

In this study, the development, validation, and application of an online SPE-UHPLC-MS/MS method for the fast and sensitive detection of 11 mycotoxins and mycotoxin metabolites in human urine samples was presented. Validation experiments revealed excellent sensitivity with limits of detection in the range of pg/mL urine. Incorporating stable isotope-labelled standards yielded high apparent recovery rates for most analytes and allowed reliable quantitation. Due to minimal sample handling during sample preparation and the automated sample extraction, high accuracy and precision was achieved, even at low urinary mycotoxin levels. Besides these aspects, the short total analysis run time of 13 min provides a major benefit of the presented online SPE procedure compared to more conventional methods such as IAC or offline SPE. While the required time effort is almost comparable to dilute and shoot approaches, the limits of detection of the newly developed method could be lowered significantly. Avoiding sample dilution and applying a higher sample volume enabled the observation of higher analyte occurrence rates in a set of 50 Zimbabwean urine samples using the online SPE procedure compared to an established dilute and shoot approach. Consequently, the presented method facilitates a high sample throughput combined with sensitive and robust evaluation of mycotoxin biomarker concentrations. Therefore, it is believed to be a powerful complement for reliable exposure assessment of mycotoxins in large sample cohorts.

## 4. Materials and Methods

### 4.1. Chemicals and Reagents

Ultrapure water of Type 1 was produced using a Purelab Flex 2 system (Veolia Water Technologies, Celle, Germany). Acetonitrile (ACN) and Methanol (MeOH) of LC-MS grade were purchased from Fisher Scientific (Schwerte, Germany). Formic acid was from Merck (Darmstadt, Germany). Ammonium acetate (for LC-MS; ≥ 99%) was obtained from VWR (Darmstadt, Deutschland). AFM_1_, CIT, and ^13^C_34_-FB_1_ were purchased from Sigma-Aldrich (Taufkirchen, Germany). DH-CIT was from AnalytiCon Discovery GmbH (Potsdam, Germany). U-[^13^C_17_]-AFM_1_ was purchased from Biopure™ Romer Labs Deutschland GmbH (Butzbach, Germany). ALT, AME, AOH, FB_1_, OTA, and ZEN were obtained via isolation and purification from fungal cultures [32,33,34,35]. α-ZEL and β-ZEL were obtained by reduction of ZEN [36]. The chemical synthesis of *d*_5_-OTA, ^13^C_3_-CIT, and ^13^C_3_-DH-CIT was performed according to Cramer et al. and Bergmann et al. [34,37]. *d*_2_-ZEN was synthetized using unlabeled ZEN [35].

The concentration of the FB_1_ stock solution was verified by quantitative nuclear magnetic resonance spectroscopy (qNMR) using Thymol (Sigma Aldrich, Taufkirchen, Germany) for internal calibration. UV spectroscopy was used to determine the concentrations of the other in-house produced standards. Stock solutions of all analytes in ACN, MeOH, or ACN/H_2_O at concentrations of 0.5–92 µg/mL were stored at -18 °C. Working solutions at 25-fold concentration of the highest calibration point in ACN/H_2_O (50:50, *v*/*v*) were prepared at each day of analysis. The stable isotope-labelled standards were combined in a separate working solution fortified at 100-fold concentration of the required concentration in the urine samples (see Table S3, Supplementary Material).

### 4.2. Urine Samples

For validation and calibration experiments the pooled urine from three female German volunteers was used. Before donating the urine, the volunteers abstained from the ingestion of cereals and cereal-based foodstuffs for 36 h to obtain urine not containing any of the analytes. Since OTA was still detectable in small amounts, the peak area was subtracted from the peak areas of all calibration points prior to linear regression. Urine samples were stored at −80 °C until analysis. Urine samples from the SHINE trial used in this study were from infants at 3, 6, 12, and 18 months after birth.

### 4.3. Sample Preparation

Urine samples were thawed to room temperature and homogenized thoroughly. An aliquot of 297 µL was pipetted into a 1.5-mL safe-lock tube followed by the addition of 3 µL of a solution containing the stable isotope-labelled standards. After vigorous shaking using a vortex shaker, the samples were centrifuged for 10 min at 15,000× *g*. Approximately 240 µL of the supernatant were subjected to online SPE-UHPLC-MS/MS analysis. 

### 4.4. Online SPE-UHPLC-MS/MS Method

Analysis was carried out using a 1260 Infinity LC system (Agilent, Waldbronn, Germany) coupled to a QTRAP 6500 mass spectrometer (SCIEX, Darmstadt, Germany). For loading the online solid phase extraction (SPE) column, an additional external pump (LaChrom HPLC Pump L-7100, Merck Hitachi, Darmstadt) was employed. A six-port column switching valve in the 1260 Infinity Thermostatted Column Compartment enabled the online sample clean-up followed by chromatographic separation of the analytes on the analytical column. An Oasis^®^ HLB column (2.1 × 20 mm, 5 µm; Waters GmbH, Eschborn, Germany) and H_2_O containing 0.1% formic acid and 5 mM ammonium acetate as a mobile phase was used to preconcentrate the analytes and remove matrix compounds after injecting 100 µL of the urine sample. The flow rate for loading and washing of the online SPE column was set to 1 mL/min. After 2 min, the valve was switched for elution of the analytes from the online SPE column in the back-flush mode and chromatographic separation on a Nucleodur C_18_ Gravity-SB column (2.0 × 75 mm, 1.8 µm) equipped with a precolumn of the same material (4.0 × 2.0 mm) (both Macherey-Nagel, Düren, Germany). The mobile phases for gradient elution consisted of ACN/H_2_O (95/5, *v*/*v*) (A) and H_2_O/ACN (95/5, *v*/*v*) (B) both containing 0.1% formic acid and 5 mM ammonium acetate. At a flow rate of 450 µL/min, the analytical column was equilibrated at initial conditions of 15% A while loading and washing the online SPE column. After valve switching, the conditions were maintained for another minute followed by increasing the percentage of A to 75% at 9.3 min. Within 0.1 min, the content of A was increased to 100% and kept constant until 11.4 min. Subsequently, the percentage of A was decreased back to 15% within 0.1 min and held for 1.5 min. During the analysis, which was carried out in a total run time of 13 min, the column oven temperature was set to 45 °C. The column switching valve changed back to the load/wash position at 12.1 min allowing for the equilibration of the online SPE column with the loading solvent prior to the next injection. Moreover, a diverter valve at the mass spectrometer was incorporated in order to discard the first 4 min of each run.

Electrospray ionization (ESI) was carried out at ion spray voltages of 5500 V in positive and −4500 V in the negative ionization mode. The source temperature was set to 500 °C. Curtain gas, nebulizer gas, and heating gas were set to 40 psi, 45 psi, and 55 psi, respectively, and the collision gas was set to “medium”. Analyte detection was performed in scheduled multiple reaction monitoring (sMRM) with a target scan time of 150 ms and detection windows of at least 30 sec width. MRM transitions as well as the respective declustering potential (DP), collision energy (CE), and collision cell exit potential (CXP) were optimized by means of direct injection of neat standard solutions. At least two MRM transitions for each analyte were monitored to assure identification. The detailed MS/MS parameters can be found in Table S1 (Supplementary Material). The entrance potential was set to 10 V for all analytes monitored in the positive ionization mode and -10 V for all analytes in the negative ionization mode. Analyst 1.6.2 Software was employed for data acquisition and data processing was performed using MultiQuant 3.0.3.

### 4.5. Method Validation

In the course of an extensive in-house validation, method performance was determined with regard to limits of detection (LOD), limits of quantitation (LOQ), linearity, and recovery, as well as precision and accuracy during intra- and interday repeatability. Estimation of LOD and LOQ was based on spiking of blank urine with all analytes at 12 concentration levels in the range of the expected values. The MRM transition with the highest S/N ratio was chosen as a quantifier for calculation of LOD at an S/N of 3 and calculation of LOQ at an S/N of 10. The final working range was specified covering two decimal powers at eight concentration levels from the respective LOQ of each analyte. In order to determine the apparent recovery (R_A_) as well as intra- and interday repeatability, the stable isotope-labelled standards were added to the spiked urine samples at medium concentrations according to the working range (see Table S3, Supplementary Material). Calculation of R_A_ was based on division of the slopes of matrix calibrations by calibrations in pure H_2_O following Matuszewski et al. [38] considering the peak area ratios of analytes to internal standards. To achieve highest accuracy in the lower concentration range, 1/x-weighted linear calibration functions were applied to all analytes. For ALT, AME, AOH, α-ZEL, and β-ZEL, no stable isotope-labelled standards were available. Therefore, α-ZEL and β-ZEL were referred to *d*_2_-ZEN, whereas calculations for ALT, AME, and AOH were carried out using the analyte peak areas instead of peak area ratios of analyte to internal standard. Linearity of the calibration curves was confirmed considering the regression coefficients as well as Mandel’s fitting test. All experiments to determine LOD, LOQ, and R_A_ were performed in triplicate. 

Intraday repeatability was evaluated by spiking of blank urine at low, medium, and high concentration levels followed by online SPE-UHPLC-MS/MS analysis 12 times. For assessing the interday repeatability, the previously described urine samples were analyzed in quadruplicate on three different days. The relative standard deviations (RSDs) were calculated in order to estimate the method’s precision, and the percentage recovery of the spiked concentration was considered for evaluation of the accuracy. 

To monitor the method performance during the analysis of urine samples from Zimbabwe, the abovementioned fortified urine was used as quality control samples and measured in each batch of 25 samples.

### 4.6. Analysis of Urine Samples Using a Dilute and Shoot Method

In addition to the analysis of the Zimbabwean urine samples employing the newly developed online SPE-UHPLC-MS/MS method, the sample set was subjected to a frequently used dilute and shoot (DaS) approach for comparative purposes. To that end, urine sample were allowed to reach room temperature, following homogenization and centrifugation for 5 min at 15,000× *g*. Subsequently, an aliquot of 11.1 µL was diluted with 100 µL of H_2_O/ACN/formic acid (95/5/0.5; *v*/*v*/*v*). The HPLC-MS/MS parameters were chosen according to Gerding et al. [8,10].

## Figures and Tables

**Table 1:** Performance characteristics of the developed online SPE-UHLPC-MS/MS method.

Analyte	LOD ^a^ [ng/mL urine]	LOQ ^b^ [ng/mL urine]	Working Range [ng/mL urine]	R^2 c^	R_A_^d^ [%]	Intraday ^f^		Interday ^g^	
						Accuracy [%]	RSD ^h^ [%]	Accuracy [%]	RSD ^h^ [%]
AFM_1_	0.0070	0.025	0.025–2.5	0.999	101	112/99	5/2	108/97	7/5
ALT	0.21	0.69	0.70–70	0.999	38 ^e^	116/97	4/1	107/94	8/4
AME	0.020	0.040	0.040–4.0	0.984	78 ^e^	179/133	9/7	172/117	11/14
AOH	0.27	0.91	0.90–90	0.993	42 ^e^	140/107	5/3	140/103	6/6
CIT	0.09	0.32	0.30–30	0.999	98	114/100	6/2	104/98	7/5
DH-CIT	0.07	0.23	0.20–20	0.998	96	110/98	10/3	109/97	6/6
FB_1_	0.0042	0.014	0.014–1.4	0.999	147	119/114	13/10	134/128	18/11
OTA	0.0036	0.012	0.012–1.2	0.999	91	120/104	9/5	112/104	11/6
ZEN	0.010	0.035	0.035–3.5	0.992	103	119/117	16/6	101/116	25/8
α-ZEL	0.05	0.15	0.15–15	0.993	86	123/118	13/5	115/117	20/5
β-ZEL	0.05	0.15	0.15–15	0.997	80	95/104	10/4	94/110	27/8

^a^ Limit of detection, determined at S/N = 3; ^b^ Limit of quantitation, determined at S/N = 10; ^c^ Regression coefficient; ^d^ Apparent recovery; ^e^ Calculations without stable isotope-labelled standards; ^f^ Determined at lowest and medium calibration point, *n* = 12; ^g^ Determined at lowest and medium calibration point, *n* = 4 on three different days; ^h^ relative standard deviation.

**Table 2:** Limits of detection achieved with the presented online SPE approach and published methods for multi-mycotoxin analysis in human urine.

Sample Preparation	Online SPE	DaS ^a^	DaS	DUS ^b^	SPE ^b^	SPE ^b^	SPE + IAC ^b^	Direct method/IAC	QuEChERS	LLE + SPE
	LOD [ng/mL urine]									
AFM_1_	0.0070	0.06	0.05	—	—	0.0003	0.06	0.002 ^c^	—	0.01
ALT	0.21	3.0	—	—	0.02	—	—	—	—	—
AME	0.020	0.3	—	—	0.001	—	—	—	0.5	—
AOH	0.27	1.5	—	—	0.01	0.01	—	—	0.4	—
CIT	0.09	1.5	—	—	—	0.003	—	0.001 ^c^	0.5	2.88
DH-CIT	0.07	0.2	—	—	—	0.003	—	0.01	—	—
FB_1_	0.0042	1.0	0.5	0.16	0.02	0.001	0.05	0.05	0.21	0.05
OTA	0.0036	0.01	0.05	0.004	0.01	0.0003	0.01	0.001 ^c^	0.01	0.03
ZEN	0.010	0.3	0.4	0.09	—	0.001	—	0.02	0.20	1.24
α-ZEL	0.05	0.5	0.5	0.17	—	0.003	0.8	0.05	0.61	0.61
β-ZEL	0.05	0.6	0.5	0.26	—	0.001	2.2	0.05	0.91	1.1
Reference	This method	[10]	[9]	[11]	[12]	[13]	[14]	[15]	[16]	[30]

DaS: dilute and shoot; DUS: dried urine spots; SPE: solid phase extraction; IAC: immunoaffinity chromatography; QuECHERS: Quick, Easy, Cheap, Effective, Rugged, Safe; LLE: liquid-liquid extraction. ^a^ Currently used in-house method based on the parameters published in [8,10]; ^b^ Including enzymatic hydrolysis; ^c^ IAC.

**Table 3:** Summarized results of analyte occurrence and concentrations after applying the online SPE-UHPLC-MS/MS method for the analysis of human urine samples from Zimbabwe (*n* = 50, duplicate analysis each).

			Concentration [ng/mL]			
Analyte	Positive Samples % (n)	Quantitated Samples % (n)	Mean ^a^	Median ^a^	Maximum	SD ^b^ Quantitated Samples
AFM_1_	18 (9)	14 (7)	0.18	0.064	0.87	0.31
AME	2 (1)	2 (1)	—	—	0.11	—
CIT	14 (7)	8 (4)	1.5	1.4	2.3	0.57
DH-CIT	18 (9)	12 (6)	0.85	0.86	1.3	0.42
FB_1_	100 (50)	100 (50)	0.68	0.29	4.6 ^c, d^	0.95
OTA	38 (19)	6 (3)	0.060	0.044	0.11	0.046
ZEN	10 (5)	8 (4)	0.23	0.11	0.40	0.14
α-ZEL	2 (1)	<LOQ	—	—	—	—

^a^ Means and median were calculated from samples with concentrations > LOQ; ^b^ Standard deviation; ^c^ Value was calculated by extrapolation of the calibration curve; ^d^ Minimum concentration 0.032 ng/mL.

## Data Availability

Additional data is available in the Supplementary Material or upon request to the corresponding author. Data from the SHINE trial will be publicly accessible at http://ClinEpiDB.org (MRCZ approval on 30 May 2012, JHU approval on 22 February 2012).

## Supplementary Materials

The following are available online at https://www.mdpi.com/article/10.3390/toxins13060418/s1, Table S1: sMRM parameters of the validated online SPE-UHPLC-MS/MS-method, Table S2: Accuracy and relative standard deviations during intra- and interday repeatability at the second highest calibration level, Table S3: Concentrations of the analytes and stable isotope-labelled standards in the calibration solutions.
