# Supplementary material for: Determination of Urinary Mycotoxin Biomarkers Using a Sensitive Online Solid Phase Extraction-UHPLC-MS/MS Method
Source: Toxins (Basel). 2021 Jun 11;13(6):418. doi: 10.3390/toxins13060418 (PMC8230879; doi:10.3390/toxins13060418)
Supplement: Supplementary file 1 [file toxins-13-00418-s001.zip › toxins-1214205-supplementary.pdf]

Determination of Urinary Mycotoxin Biomarkers Using a Sensitive Online Solid Phase Extraction-UHPLC-MS/MS Method

Jessica Schmidt, Benedikt Cramer, Paul C. Turner, Rebecca J. Stoltzfus, Jean H. Humphrey, Laura E. Smith and Hans-Ulrich Humpf

Table S1. sMRM parameters of the validated online SPE-UHPLC-MS/MS method.

Analyte	Q1 mass [Da] [a]	Q3 mass [Da] [a]	Expected Retention Time [min]	Window [sec]	DP [V] [b]	CE [V] [c]	CXP [V] [d]
AFM_1	329.1 $[M+H]^+$	229.0	5.7		41	53	15
	329.1 $[M+H]^+$	273.0	5.7		41	33	15
ALT	293.1 $[M+H]^+$	239.0	6.1		30	27	12
	293.1 $[M+H]^+$	139.0	6.1		30	84	12
AME	271.0 $[M-H]^-$	213.1	8.5		−44	−45	−20
	271.0 $[M-H]^-$	211.1	8.5		−44	−50	−20
AOH	257.0 $[M-H]^-$	147.1	7.0		−80	−38	−11
	257.0 $[M-H]^-$	157.1	7.0		−80	−36	−11
CIT	251.1 $[M+H]^+$	215.0	6.9	60	75	35	13
	251.1 $[M+H]^+$	205.0	6.9	60	75	36	13
DH−CIT	267.1 $[M+H]^+$	203.1	6.6	45	51	39	14
	267.1 $[M+H]^+$	231.1	6.6	45	51	32	14
FB_1	722.4 $[M+H]^+$	334.2	6.4		41	55	18
	722.4 $[M+H]^+$	352.2	6.4		41	51	20
OTA	404.1 $[M+H]^+$	221.0	8.5		35	48	11
	404.1 $[M+H]^+$	102.0	8.5		35	88	11
	404.1 $[M+H]^+$	239.0	8.5		35	31	11
ZEN	317.2 $[M-H]^-$	175.0	8.6		−140	−32	−11
	317.2 $[M-H]^-$	131.0	8.6		−140	−38	−11
α−/β−ZEL	319.2 $[M-H]^-$	174.1	7.5	60	−160	−34	−15
	319.2 $[M-H]^-$	160.1	7.5	60	−160	−39	−15
$^{13}C_{17}$−AFM_1	346.3 $[M+H]^+$	242.2	5.7		41	53	15
	346.3 $[M+H]^+$	288.1	5.7		41	33	15
$^{13}C_3$−CIT	254.1 $[M+H]^+$	218.0	6.9	45	75	35	13
	254.1 $[M+H]^+$	208.0	6.9	45	75	36	13
$^{13}C_3$-DH−CIT	270.1 $[M+H]^+$	206.1	6.6		54	39	14
	270.1 $[M+H]^+$	234.1	6.6		54	32	14
$^{13}C_{34}$−FB_1	756.1 $[M+H]^+$	356.3	6.4		41	55	18
	756.1 $[M+H]^+$	374.3	6.4		41	51	20
d_5−OTA	409.1 $[M+H]^+$	221.0	8.5		35	48	11
	409.1 $[M+H]^+$	102.0	8.5		35	88	11
	409.1 $[M+H]^+$	239.0	8.5		35	31	11
d_2−ZEN	319.2 $[M-H]^-$	177.0	8.6		−140	−32	−11
	319.2 $[M-H]^-$	133.0	8.6		−140	−38	−11

[a] Quantifier above, qualifier below; [b] declustering potential; [c] collision energy; [d] collision cell exit potential

Table S2. Accuracy and relative standard deviations determined during intra− and interday repeatability at the second highest calibration level.

Analyte	Intraday [a]		Interday [b]	
	Accuracy [%]	RSD [c] [%]	Accuracy [%]	RSD [c] [%]
AFM_1	103	3	101	4
ALT	99	1	97	3
AME	134	5	117	15
AOH	110	3	104	5
CIT	103	2	102	4
DH−CIT	103	5	103	6
FB_1	117	8	128	10
OTA	106	4	105	5
ZEN	117	5	116	4
α−ZEL	115	6	115	6
β−ZEL	103	7	110	10

[a] $n = 12$; [b] $n = 4$ on three different days; [c] relative standard deviation.

Table S3. Concentrations of the analytes and stable isotope−labelled standards in the calibration solutions.

Analyte	Concentration Levels in Calibration Solutions [ng/mL]									
	1	2	3	4	5	6	7	8	Int. Standard	1–8
AFM_1	0.025	0.063	0.13	0.19	0.25	0.63	1.3	2.5	$^{13}C_{17}$−AFM_1	0.20
ALT	0.70	1.8	3.5	5.3	7.0	18	35	70	—	—
AME	0.040	0.10	0.20	0.30	0.40	1.0	2.0	4.0	—	—
AOH	0.90	2.3	4.5	6.8	9.0	23	45	90	—	—
CIT	0.30	0.75	1.5	2.3	3.0	7.5	15	30	$^{13}C_3$−CIT	3.0
DH−CIT	0.20	0.50	1.0	1.5	2.0	5.0	10	20	$^{13}C_3$-DH−CIT	2.0
FB_1	0.014	0.035	0.070	0.11	0.14	0.35	0.70	1.4	$^{13}C_{34}$−FB_1	0.14
OTA	0.012	0.030	0.060	0.090	0.12	0.30	0.60	1.2	d_5−OTA	0.12
ZEN	0.035	0.088	0.18	0.26	0.35	0.88	1.8	3.5	d_2−ZEN	0.35
α−ZEL	0.15	0.38	0.75	1.1	1.5	3.8	7.5	15	d_2−ZEN	0.35
β−ZEL	0.15	0.38	0.75	1.1	1.5	3.8	7.5	15	d_2−ZEN	0.35
